# Extracting chemical–protein relations using attention-based neural networks

**DOI:** 10.1093/database/bay102

**Published:** 2018-10-08

**Authors:** Sijia Liu, Feichen Shen, Ravikumar Komandur Elayavilli, Yanshan Wang, Majid Rastegar-Mojarad, Vipin Chaudhary, Hongfang Liu

**Affiliations:** 1 Department of Health Sciences Research, Mayo Clinic, Rochester, MN, USA; 2 Department of Computer Science and Engineering, University at Buffalo, Buffalo, NY, USA; 3 University of Wisconsin-Milwaukee, Milwaukee, WI, USA

## Abstract

Relation extraction is an important task in the field of natural language processing. In this paper, we describe our approach for the BioCreative VI Task 5: text mining chemical–protein interactions. We investigate multiple deep neural network (DNN) models, including convolutional neural networks, recurrent neural networks (RNNs) and attention-based (ATT-) RNNs (ATT-RNNs) to extract chemical–protein relations. Our experimental results indicate that ATT-RNN models outperform the same models without using attention and the ATT-gated recurrent unit (ATT-GRU) achieves the best performing micro average F1 score of 0.527 on the test set among the tested DNNs. In addition, the result of word-level attention weights also shows that attention mechanism is effective on selecting the most important trigger words when trained with semantic relation labels without the need of semantic parsing and feature engineering. The source code of this work is available at https://github.com/ohnlp/att-chemprot.

## Introduction

The current scientific discovery in the biomedical domain highly depends on knowledge resources that catalog scientific findings in a computable format to facilitate data analysis and interpretation due to the advancement of high-throughput technologies. However, valuable information of scientific findings is generally embedded in literature and it is very expensive and time-consuming to acquire such information from literature manually (1, 2). In the past decade, natural language processing (NLP) has been applied to accelerate the acquisition process with reasonable success (3, 4). Previous BioCreative challenges have produced named-entity recognition (NER) tools (5–8) for extracting and normalizing a wide range of biomedical entities with good performance. Recent subsequent NLP challenges have started to focus on the extraction of relations among those entities. The chemical–protein interactions (ChemProt) task in BioCreative VI aims to automatically extract the interaction information between chemical compounds and genes/proteins as interactions between chemical compounds and gene products are essential for understanding metabolism, signaling and drug treatment (9).

Deep learning approaches have been extensively studied and achieved state-of-the-art performances in various NLP tasks such as NER (10–12) and relation extraction (13–16). Despite many empirical successes demonstrated via quantitative evaluation metrics, deep learning models have long been challenged as ‘black boxes’. It is mainly due to the difficulty in tracing the prediction of deep learning models back to important explicit features. Therefore, it is of interest and importance to show the effectiveness of deep learning models on extracting explicit features to unveil how deep neural network models work. In biomedical relation extraction contexts, trigger words, which are the words appearing in the context of biomedical entities and directly indicating the existence of semantic relations, are widely used as input features of various text mining methods (17–21). Attention mechanism (22), proposed from the intuition of visual attentions of human to emphasis the relatively important part of the input data, has been shown to improve model performance and enhance the model interpretability via incorporating the attention information into deep learning (23). Here, we present our approach for the ChemProt task using attention-based (ATT-) neural networks and demonstrate the strength of ATT-models in performance by comparing with other deep learning approaches and their interpretability by analyzing the word-level attention weights.

The paper is organized as follows. We first briefly review the related work. The proposed methods for the ChemProt task including the overall architecture and the detailed learning strategy are described next. We then present our experimental results with different perspectives of evaluation, followed by the analysis of the trained attention weights. Finally, we discuss the limitations of our methods and conclude the paper with several future directions.

## Related work

In the general domain, deep neural networks (DNNs) have been utilized widely in relation extraction tasks. Various DNN models were explored for relation extraction on the SemEval 2010 Task 8 benchmark. For example, Zeng *et al.* (24) proposed convolutional neural network (CNN) using position embedding for relation extraction. Xu *et al.* (25) used dependency and position embeddings with long short-term memory (LSTM) model and showed their learning strategy significantly outperforms the recurrent neural network (RNN) methods with extensive features including part-of-speech (POS) tags, NER results and WordNet features.

In the biomedical domain, various relation extraction tasks such as protein–protein interactions (26, 27), drug–drug interactions (28) and chemical–disease interactions (29) have been investigated in prior shared tasks in the biomedical domain. Various machine learning-based methods including supervised machine learning methods (30, 31), pattern clustering (32) and topic modeling (33) were used before deep learning models became dominant among the recent advances. Besides conventional DNN models (34, 35), dependency (15, 36) and character level (16) information have been used to enhance the models with improvement over their baselines. Recently, the attention mechanism on top of DNN models has shown promise in various NLP tasks, such as machine translation (23), question answering (37), document classification (38) as well as relation extraction. Several studies have used a sentence-level ATT-model for relation extraction and employ CNNs to embed the semantics of sentences (39, 40). Shen *et al.* (41) investigated multi-level attention CNNs to discern patterns in heterogeneous contexts.

In this work, we investigate the ATT-neural network for the ChemProt task and demonstrate the effectiveness of attention mechanism on selecting importance and informative word-level information without using external knowledge and extensive feature engineering, thus can be a generalizable model for relation extraction.

## Materials and methods

**Figure 1:**
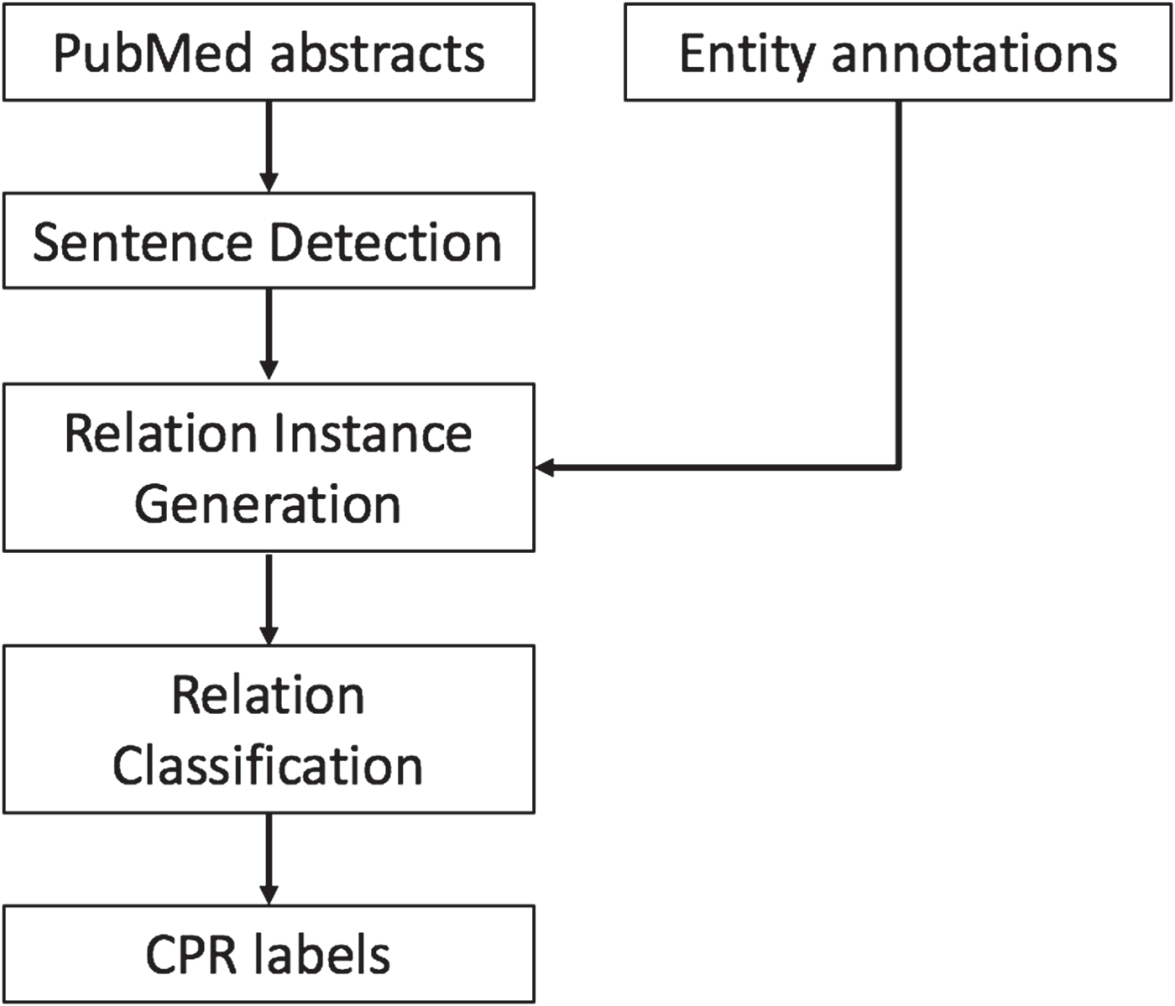
Overview of the system workflow.

The system architecture of our methods is illustrated in Figure 1. Given the raw text of ChemProt-related articles and the annotated chemical protein/gene entity mentions, we model the relation extraction problem as a relation classification problem among all the potential ChemProt relation (CPR) pairs. We first divide the text into sentences. Then the entity annotations are aligned with each sentence. The sentence and the set of entities within the sentence are then used to generate relation instances. The word embedding and position embedding features are extracted for each relation instance as the input of our proposed neural network models. The final output is the prediction of labels of each relation instance, including the pairs with no CPR. Eventually, only the predicted relations with CPR types of interest are extracted in the required format for the final evaluation.

### Sentences detection and relation instance generation

In this study, we only consider the relations between entities appearing in the same sentence. We use Punkt sentence detector in Natural Language Toolkit (42, 43) to detect sentence boundaries. The title of each article is regarded as the first sentence of the abstract, and it is not treated separately.

**Figure 2:**
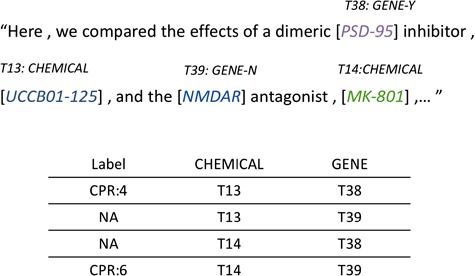
Relation instance generation from annotated entities within sentence.

For each potential ChemProt pair in the sentence, we assign a relation label ‘NA’ for the pair without annotated gold standard annotation provided by the challenge organizers. Here we consider the relations other than the five evaluated types as negative relations (CPR 1, 2, 7, 8 and 10). An example of how relation instances are generated from sentences is shown in Figure 2. There are two chemical mentions, ‘*UCCB01-125*’ and ‘*MK-801*’, and two gene mentions, ‘*PSD-95*’ and ‘*NMDAR*’, in the sentence, yielding four candidate pairs with two of them as positive CPRs.

### Input representation

In our proposed system, the input to the DNN models is expected to be low-dimension semantic word-level vectors.

As a preliminary experiment, we test two different methods to represent the pre-annotated entities. In the first method, the raw word in the sentences are directly sent to the word embedding model to retrieve the word embeddings, regardless if it is an annotated word as part of an entity. The limitation of this method is that a lot of chemical, gene and protein entities may not be found from the pre-trained word-embedding model. Besides, many of the entity mentions are phrases (e.g. ‘human ether-a-go-go-related gene (HERG) potassium channel’), which makes it challenging to obtain semantic vector representations based on word-level embeddings. Therefore, we replace all the chemical and gene/protein entity mentions by their entity types. Specifically, for chemical entity mentions, all the words of the entity are replaced by the word ‘chemical’, and all the gene and protein entities are replaced by ‘gene’. By doing so, the number of out-of-vocabulary words significantly decreases, and the entity mentions with multiple words get properly handled.

There are two kinds of features we used as the input of the DNN models:

*Word embeddings*. We use 300-dimension pre-trained Glove-6B model (https://nlp.stanford.edu/projects/glove/). Our preliminary experiments show that the 300-dimension Glove-6B outperforms the word embedding models we trained by continuous bag of words (CBOW) from PubMed (44). If a word cannot be found from the word embedding model, the embedding will be generated randomly and the generated embedding will be appended into the model.

**Figure 3:**
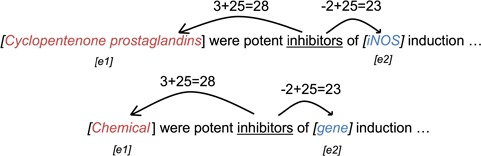
Example of position embedding indices of the word ‘inhibitors’ using raw words (upper) and entity labels (lower).

*Position embeddings*. We follow the method by Zeng *et al.* (24) to generate the position embedding of the entities in each narrative sentence. The position embedding is generated based on the relative distances of words to the entities. An example of relative distance is shown in Figure 3. The distances are then shifted by an arbitrary offset to map the distances to positive integers. The shifted distance $d=25$ is then used as the index of the position embeddings. The position embeddings are then jointly trained during the training phase.

For each relation instance, there will be two position embeddings for each word from two entities for chemical and protein entities, respectively. The two position embeddings are concatenated to the word embeddings of the word as the input to the neural network models. In this study, we use two 50-dimensional position embeddings and 300-dimensional word embeddings, yielding a 400-dimensional feature vector for each word.

### DNN models

We experiment with three DNN models.

**Figure 4:**
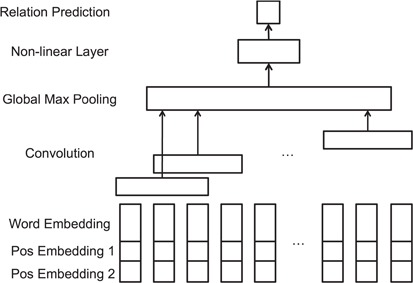
CNN model for relation extraction.

*CNNs.* To demonstrate the effectiveness of our proposed ATT-model, we first developed a relation extraction model using CNN as baseline, which is one of the most widely used DNN model. The CNN model for relation extraction is built according to Zeng *et al.* (24). The model architecture is shown in Figure 4. The convolutional layer can capture contextual information of filters of a pre-defined filter length. The convolutional filters are expected to generate high-level local features from the input vector representations. The output of the convolutional layer is then forwarded to the Global Max-pooling layer, where the maximum values of each filter outputs are pooled and concatenated for relation classification.

**Figure 5:**
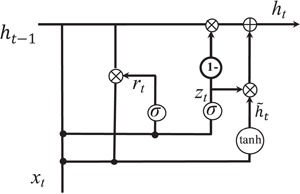
Illustration of gated recurrent units.

*Gated recurrent unit.* While CNN can capture the local patterns in the convolutional space as larger structures, RNN models are designed to learn the patterns across time of given sequences. We investigate the usage of RNN for relation extraction. RNN models the sentence into a sequence of vectors. In this paper, we would like to test a different RNN unit, gated recurrent unit (GRU) for the task. GRU was first proposed by Cho *et al.* (45). The intuition behind GRU unit is similar to LSTM regarding the gating mechanism to combine the updates and current input into each RNN unit. Previous model comparison for other deep learning tasks showed that there is no obvious winner between LSTM and GRU (46).

We follow the formulation of GRU used by Chung *et al.* (46). A GRU can be illustrated as Figure 5. Using ${x}_t$ to denote input vector, $W$ to denote the transform matrix of the inputs, $U$ to denote the transform matrix of hidden states and $b$ as the bias, the output of the GRU ${h}_t$ can be calculated as

\begin{align*} {z}_t=&\ \sigma \left({W}_z{x}_t+{U}_z{h}_{t-1}+{b}_z\right),\\{r}_t=&\ \sigma \left({W}_r{x}_t+{U}_r{h}_{t-1}+{b}_r\right),\\[-2pt]\tilde{h}_t=&\ \tanh \left({W}_h{x}_t+{U}_h\left({r}_t\ast{h}_{t-1}\right)+{b}_h\right),\\[-2pt] {h}_t=&\ {z}_t\ast{h}_{t-1}+\left(1-{z}_t\right)\ast \tilde{h}_t,\end{align*}

where $\sigma \left(\cdot \right)$ denotes sigmoid function and ^*^ denotes the element-wise multiplication. The ‘reset gate’ ${r}_t$ represents how much the current state is affected by the previous activation. $\tilde{h}_t$ is the hidden ‘state candidate’ of the output. The ‘update gate’, ${z}_t$, aims to decide the scale of the unit based on the previous activation, and it controls how much the output ${h}_t$ is affected by $\tilde{h}_t$.

*ATT-RNN*. Attention mechanism is proposed to emphasize the contribution of the informative neural units in the model. Instead of directly receiving the activations or outputs from consecutive RNN units, the additional attention layer overlooks all the RNN units of the input sequence and assigns different weights to each unit according to their importance. The intuition for applying ATT-model in relation extraction task is to try to assign higher weights for words that are indicators or trigger words of specific semantic relations.

**Figure 6:**
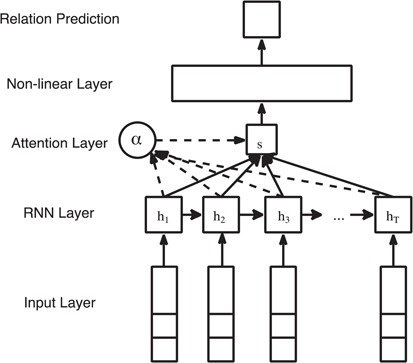
Attention-based RNN for relation extraction.

We use the equations inspired by Luong *et al.* (23) to calculate attention weights for each word in a sentence. The ATT-RNN for relation extraction is illustrated in Figure 6. The activations of the RNN units is denoted as $h=[{h}_1,{h}_2,\\\dots, {h}_T]$, where $T$ is the sentence length. Given word representation as $w$ and the activations of previous RNN units ${h}_t$, we define the hidden weight matrix of the attention layer as ${u}_t$ and word-level importance vector ${u}_w$, which is a trainable variable. The relation representation vector *s* is the weighted sum of RNN outputs $h$ and the attention weights $\alpha$.

\begin{align*} {u}_t=&\ \tanh \left({W}_w{h}_t+{b}_w\right),\\{\alpha}_t=&\ \frac{\exp \left({u}_t^T{u}_w\right)}{\sum_t\exp \left({u}_t^T{u}_w\right)},\\ s=&\ {\sum}_t{\alpha}_t{h}_t,\end{align*}

where ${W}_w$ and ${b}_w$ are the weight matrix and bias of the attention layer, similar to the notations of the GRU layer.

### Classification

Finally, a non-linear function can be used on the max-pooling vector to predict the probability-like values of each relation label. We then select the label with the highest value from the non-linear layer as the relation label. The classification step is identical among CNN, RNN and attention models.

A non-linear layer is implemented to use a softmax function to predict CPR labels of each relation instance into $K=6$ categories: five CPR types identified in the shared task plus a class of ‘NA’ as ‘not related’. Taking the output of either convolutional layer, RNN layer or attention layer ${h}^{\ast }$ as input of this non-linear layer, the predicted probability of each label $k\in \left\{1,\dots, 6\right\}$ of given input $\hat{p}\left(y=k|x\right)$ can be obtained by

$$ \hat{p}\left(y=k|x\right)=\frac{\exp \left({W}_{kc}h+{b}_{kc}\right)}{\sum_{j=1}^K\exp \left({W}_{jc}h+{b}_{jc}\right)}. $$

$ W_{\cdot{c}}, b_{\cdot{c}}, h_{\cdot{c}}$ are trainable parameters of the fully connected layer.

Then, denoting the predicted discrete class labels as $\hat{y}$, we have

$$ \hat{y}={\arg\max}_y\ \hat{p}\left(y=k|x\right), $$

which is regarded as the predicted CPR label of the relation instance for the evaluation.

### Evaluation

The system performance is evaluated by the official evaluation package provided by the task organizers (http://www.biocreative.org/media/store/files/2017/evaluation-kit.zip) via precision, recall and standard micro-average F1 score defined in the following:

\begin{align*} &Precision=\ \frac{TP}{TP+ FP}\\ &Recall=\ \frac{TP}{TP+ FN}\\ &F1=\ \frac{2\cdot Precision\cdot Recall}{Precision+ Recall} \end{align*}

Here, TP (true positive) denotes the number of correctly detected positive relation instances, FP (false positive) denotes the number of relations ‘NA’ in the gold standard but are predicted as one of the positive relations by the DNN model, false negative (FN) denotes the number of positive instances that are not detected by the model.

## Results

### Data set

The ChemProt corpus consists of 4966 PubMed abstracts with 126 457 annotated chemical and protein entities. The relations were annotated with 10 CPRs. According to the shared task description, only 5 out of 10 semantic relation types would be evaluated. Therefore, we focused only on the relation groups included in the official evaluation (CPR 3, 4, 5, 6 and 9). The details and subgroups of in each relation type are shown in Table 1.

**Table 1:** Relation types for ChemProt

Relation label	Subgroups
CPR:3	upregulator, activator, indirect upregulator
CPR:4	downregulator, inhibitor, indirect downregulator
CPR:5	agonist, agonist-activator, agonist-inhibitor
CPR:6	antagonist
CPR:9	substrate, product of,

**Table 2:** ChemProt corpus statistics

Data set	No. of docs	Average no. of entities	No. of positive relations	No. of all relation instances
Training	1020	25.247	4157	15 842
Development	612	25.436	2416	9759
Test	800	25.536	3458	13 095

**Table 3:** Hyperparameter setting for CNN and ATT-RNN models

Hyperparameter	Optimal value	Tested values
Batch size	64	[16, 32, 64, 128, 256]
Number of CNN filters	100	[30, 50, 100, 150, 200]
Filter length	3	[3, 4, 5]
RNN dimension	128	[32, 64, 128, 256, 512]
Learning rate	0.001	[0.01, 0.005, 0.001, 0.0005, 0.0001]

**Table 4:** Official submissions to BioCreative VI ChemProt challenge

Run ID	Model	Train vs dev			Train vs test		
		Precision	Recall	F1 score	Precision	Recall	F1 score
1	CNN token	0.459	0.456	0.457	0.477	0.437	0.456
2	CNN entity	0.497	0.448	0.471	0.507	0.430	0.465
3	ATT-GRU token	0.470	**0.522**	0.494	0.484	**0.491**	0.488
4	ATT-GRU entity	**0.512**	0.501	**0.506**	**0.530**	0.463	**0.494**

Table 2 shows the corpus statistics of the training, development and testing data sets, including the number of documents in the data set, the average number of entities per document (abstract) and the average number of positive relations per document.

### Parameter settings

The models are implemented using Keras (https://keras.io/) with Tensorflow (https://github.com/tensorflow/tensorflow) backend. The models are trained using Adam optimizer on the loss function of sparse categorical cross entropy. Dropout was applied to the non-linear layers to prevent overfitting (47), and the dropout rate was set to 0.5. Table 3 lists the hyperparameters tested and their optimal values. The choices of hyperparameters need to be made carefully with both the support of rationale and experimental results. Learning rate and batch size together control the converge of the model training, the effect of which are evaluated based on system performance in F1 score. The number of RNN dimension directly affects the size of the model, namely the number of trainable parameters. If the RNN dimension is too large, the model will fail to converge due to the lack of sufficient training samples. If the RNN dimension is set too small, the model may not be capable to capture existing patterns. The optimal hyperparameter values are selected based on grid search on the vector space using the development set.

### Experimental results

We submitted four runs to the official BioCreative VI ChemProt task evaluation trained using the training set:

**Run 1:** CNN with raw tokens as input, without replacing annotated entity tokens.

**Run 2**: CNN with raw tokens replaced as labels.

**Run 3**: ATT-GRU raw tokens as input, without replacing annotated entity tokens.

**Run 4**: ATT-GRU with raw tokens replaced by entity labels.

Table 4 shows the system performance of each submitted run in the development and test sets. Based on our experimental results, ATT-GRU models (Run 3 and 4) outperform CNN models (Run 1 and 2) and replacing raw entity mentions by entity labels enhances the models slightly for both CNN and ATT-GRU. The best run, achieved by the ATT-GRU model with entity labels, has an F1 score of 0.494 on the test set. Replacing entity mentions by entity labels overcomes out-of-vocabulary issues associated with chemical, gene and protein tokens. The F1 scores only slightly decrease ($\le 0.012$) when evaluated on the test set comparing to those achieved on the development set which indicates our models generally do not suffer much from overfitting.

We further evaluate the DNN models more comprehensively after the test set made publicly available. We train DNN models using both the training set and the development set, i.e. a total of 1600 abstracts. Table 5 shows the post-challenge evaluation of various DNN models in the test data set. The RNN and LSTM models cannot converge using our feature set in our preliminary experiments and will fail to predict any positive relation. Therefore, we did not include them into our results. We observe an increase of F1 score from 0.496 to 0.527 for the ATT-GRU model on entity labels due to the increase of training instances. This implies that the model can be enhanced by incorporating more training data, a common characteristic for supervised machine learning systems. Note that we can add weights to each CPR type while calculating the loss function to balance the precision and recall. However, the weights are not consistent across different DNN models and adding weights does not necessarily yield better F1 scores.

**Figure 7:**
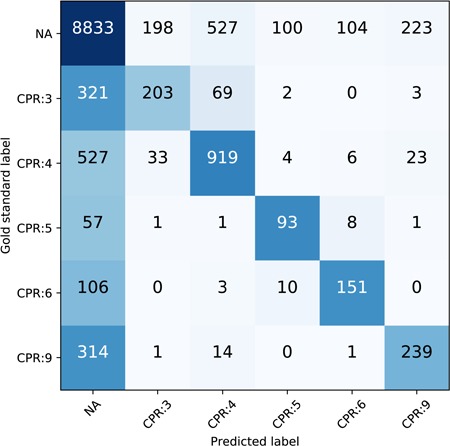
Confusion matrix of the CPR on the test set, normalized by row.

The performance breakdown of each CPR type of our best run (ATT-GRU) on the test set is shown in Table 6. The classification report is generated via scikit-learn (http://scikit-learn.org/stable). CPR:4 has the highest F1 score among all the CPR types and has the largest proportion in all relations as well. CPR:3 is one of the most difficult relation types to classify. To further illustrate the prevalence of CPR extraction errors, we plot the confusion matrix of the CPR classification of ATT-GRU model on the test set as shown in Figure 7. The *x* axis is the predicted label by the model while the *y* axis is the gold standard label. The numbers in each cell are the total relation instances. The color is normalized by row including the ‘NA’ instances, with darker blue indicating more instances and the lighter white indicating less instances. The confusion matrices and the classification reports of other DNN models are also provided in the supplemental material. From the confusion matrix, we can see that the major challenge of the relation classification of our proposed model is the large number of negative instances. Comparing to the misclassified relations with positive labels, more of the CPR types suffer from how to ‘detect’ the existence of the CPRs accurately. Besides that, the confusion between different relation types is relatively small compared to the positive/negative errors. 

**Table 5:** Post-challenge evaluation of DNN models using replaced entities

Model	Train vs dev			Train + dev vs test		
	Precision	Recall	F1 score	Precision	Recall	F1 score
CNN	0.497	0.448	0.471	0.546	0.434	0.483
GRU	0.494	0.446	0.469	0.532	**0.487**	0.509
ATT-RNN	**0.516**	0.404	0.445	0.522	0.445	0.481
ATT-LSTM	0.485	0.429	0.456	0.572	0.465	0.513
ATT-GRU	0.512	**0.501**	**0.506**	**0.574**	**0.487**	**0.527**

**Table 6:** Performance breakdown of ATT-GRU on the test set

Label	Support	Precision	Recall	F1 score
CPR:3	598	0.544	0.355	0.429
CPR:4	1512	0.607	0.641	0.623
CPR:5	161	0.524	0.534	0.529
CPR:6	270	0.615	0.504	0.554
CPR:9	569	0.494	0.492	0.493

### Analysis of the word-level attention weights

**Figure 8:**
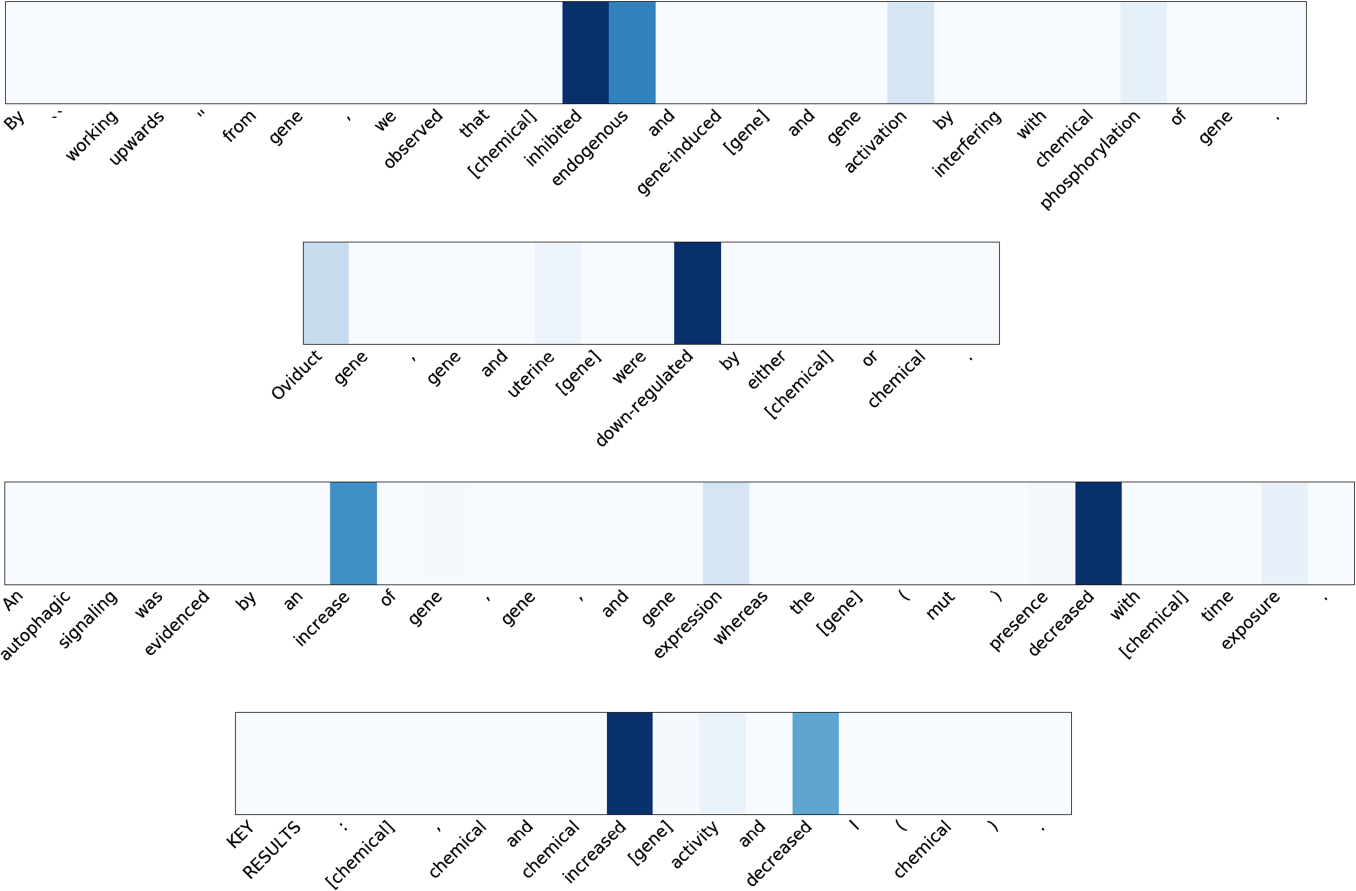
Examples of attention weight distribution with in each relation instance. The chemical and gene/protein entities are surrounded by square parenthesis (‘[ ]’). Darker background color (dark blue) on the word block indicates higher attention weights.

The most prominent advantage of using attention mechanism is that it can learn the word-level important features using the RNN outputs aligned with input words. To demonstrate the effectiveness of attention weight assignment, we have done further analysis on the attention weights trained from the training and development set using the test set, which is considered unobserved by the model. The attention weights are computed using the activations of the RNN layer and the trained parameters. Figure 8 shows examples of attention weight distribution at sentence-level. It demonstrates that the attention mechanism can highlight keywords with important indicators in semantic relations effectively.

To further demonstrate what pattern from the attention we can learn from the ChemProt corpus, we collect all the words with the largest attention in each positive instance among the test set and show the top 5 words of each CPR type in Table 7. From the top word list, we can see there are some CPR types that have high concentration of key words, such as CPR: 4, 5 and 6. Especially, in CPR: 4, all the top 5 words are the variations of “inhibitor”, which is also in the definition of CPR: 4. For CPR: 5 (agonist, agonist-inhibitor), the top three words in the sentence “agonist”, “receptor” and their plural form contributes to in total of 99.1% of the CPR: 5 relation instances, which indicates that the occurrence of “agonist” and “receptor” itself can be considered as a very strong indicator of CPR: 5 relations. Similarly, the variations of “antagonists” contributes to 88.1% in CPR: 6. It is also notable that the system is able to identify key terms like ‘enzyme’, ‘substrate’ and ‘catalyze’ for CPR: 9 highlight deeper semantic association and textual variants as relevant to the broad class substrate a central concept in enzyme kinetics.

## Discussion

The experimental results we have presented show the attention mechanism is effective for selecting the most important features when classifying semantic relations without the need of extensive feature engineering. The enhancement of ATT-models to conventional DNN models is in both the performance and interpretability.

**Table 7:** Top five attention keywords in each ChemProt relation type. The words are represented in the format of ‘word with largest attention weights in the sentence (number of the occurrence in each category, percentage of the occurrence in each category)’

CPR:3 (up-regulator, activator)	CPR:4 (down-regulator, inhibitor)	CPR:5 (agonist, agonist-inhibitor)
Expression (44, 8.8%) mRNA (32, 6.4%) Phosphorylation (30, 6.0%) Activation (26, 5.2%) Inhibited (23, 4.6%)	Inhibitor (192, 19.4%) Inhibitors (128, 12.9%) Inhibition (105, 10.6%) Inhibited (60, 6.1%) Inhibits (33, 3.3%)	Agonist (51, 45.5%) Agonists (45, 40.2%) Receptors (13, 11.6%) Receptor (2, 1.8%) Antagonism (1, 0.9%)
CPR:6 (antagonist)	CPR:9 (substrate, product of)	
Antagonist (91, 49.5%)Antagonists (60, 32.6%)Antagonism (11, 6.0%)Agonist (7, 3.8%)Receptor (4, 2.2%)	Enzyme (43, 10%)Transporter (33, 8%)Uptake (26, 6%)Catalyzes (23, 5%)Catalyzed (19, 4%)	

In the official evaluation of BioCreative VI ChemProt task, our best submission trained only on the training set without using the development set ranked 17 out of 45 submitted runs (0.494 F1 score). Two of the top three teams (Peng *et al.* (48) with an F1 score of 0.641 and Mehryary *et al.* (49) with an F1 score of 0.609) used ensembles of support vector machine (SVM) and DNNs. Peng *et al.* developed a rich feature SVM including words, POS tags, chunk types, contextual words of entities, distance, selected keywords and shortest dependency path features. CNN and RNN models were also trained on word embeddings, POS tags, IOB (Inside-Outside-Beginning) tags and position embeddings. The majority voting was done between five SVMs, five CNNs and five RNNs, which achieved the top performing run of the shared task with 0.6410 F1 score. Mehryary *et al.* used a hybrid system of Turku Event Extraction System (TEES) (50) and DNN models in the task. The feature sets of SVM-based system TEES are similar to Peng *et al.*’s. The DNN models from (49) consist of separated LSTM models trained from words, POS tags and dependency type sequences. The submitted results are obtained from an ensemble of four neural networks with different random seeds. Our proposed ATT-GRU model is favorably comparable to their top performing run of 0.5249 F1 score from DNN, while we only used the word sequence and position embedding without POS and dependency features. Corbett and Boyle (51) (0.614 F1 score) explored the application of transfer learning and pre-trained LSTM model and word embeddings from unlabeled data. They used two neural networks for the task: a pre-training network and a recognition network. The pre-training network turned out to boost the performance via training on unlabeled data and word embeddings. In comparison with those top-performing systems, our system used a single DNN model without voting/stacking mechanisms. In addition, our system did not utilize features extracted from external language resources such as POS and dependency information, as well as additional unlabeled data. We only used the annotated sequences of words and position embeddings from the data set annotation as input features. Another difference of our proposed model is that the trigger words are outcomes of trained ATT-DNN models, rather than input features used by other supervised machine learning models such as SVM.

It is also worth noting that our attention-based approach has several limitations.

Due to the limited features used as the input of the DNN layers, the proposed method may not perform well when processing sentences with multiple relation pairs. Between multiple relation instances in the same sentence, the attention weights will be slightly different among different relation instances. However, since the classification is done after the summation of hidden vector from RNN layer weighted by the attention weights, the model will not collect sufficient information to distinguish the positive relations and negative relations if there are positive relations in the sentence. This is why the attention mechanism works well on finding the keywords related to the relations from the sentence, but the performance of overall sentence classification is not as good as expected.

The word-level features are inadequate in capturing dependency information in long sentences. The redundancy of sentence structure may also be hard for flat RNN vectors to capture. For instance, in the sentence of ‘Treatment with *[CAPE]* decreased protein abundance of [*Akt*], [*Akt1*], [*Akt2*], [*Akt3*], …, but increased cell cycle inhibitor [*p27Kip*]’, there are 13 protein entities in the sentence, thus the distance between the first and the last protein entity in the sentence is too long for recurrent units to memorize and distinguish with each other. Though the attention weights correctly highlight the words ‘decrease’, ‘increase’ and ‘inhibitor’, the differences among the weights are minimal. There are two potential solutions to this diminishing information issue. Intuitively, heuristic rules can be applied as a step of pre-processing or post-processing to merge multiple entities into one for relation classification. The machine learning-based solution might be to use Abstract Meaning Representation (AMR) (52) to trim the sentence and use the structured sentence abstract, which also removes the redundancy in the sentences and proved to be effective for other biomedical relation extraction tasks (15, 53). The semantic embeddings of AMR and dependency parsing results can be used as other word-level embeddings, such as word embeddings and position embeddings in this study.

## Conclusion and future work

In this paper, we describe our proposed system for Biocreative VI Task 5: text mining ChemProt interactions. The incorporated attention layer into RNN improves both the performance and the interpretability of the original DNN models. Our experiment demonstrates that the attention-based models outperform other deep learning models without attention in the task of CPR extraction. The results of attention weight distribution and top attention words show that the attention mechanism is effective in highlighting semantic association and textual variants of CPRs when trained with labeled CPR instances without the prior domain knowledge and extensive feature engineering.

There are some directions to extend this work to a more comprehensive neural-based relation understanding framework. We would like to see if an external knowledge base can be used to improve our machine learning-based system, which is dependent on the provided corpus. Since the word embedding plays a critical role in representing word-level information in CPR sentences, we would also like to investigate more options of word embeddings using external resources (51). We are also interested in exploring how to apply the word-level attention weights directly to the relation classification tasks using pattern generation and sub-language analysis techniques.

## Supplementary Material

Supplementary Data

## Notes

**Database URL:** https://github.com/ohnlp/att-chemprot
