# Supplementary material for: Extracting chemical–protein relations using attention-based neural networks
Source: Database (Oxford). 2018 Oct 8;2018:bay102. doi: 10.1093/database/bay102 (PMC6174551; doi:10.1093/database/bay102)
Supplement: Supplementary Data [file supplement_material.docx]

Supplement Material

Confusion metrics and classification reports from various DNN models. Both of the reports are generated using scikit-learn.

# CNN

|  | Predicted | | | | | | |
| --- | --- | --- | --- | --- | --- | --- | --- |
| GS |  | NA | CPR:3 | CPR:4 | CPR:5 | CPR:6 | CPR:9 |
|  | NA | 8946 | 201 | 490 | 46 | 52 | 250 |
|  | CPR:3 | 322 | 207 | 60 | 0 | 3 | 6 |
|  | CPR:4 | 603 | 53 | 824 | 0 | 3 | 29 |
|  | CPR:5 | 90 | 3 | 5 | 62 | 0 | 1 |
|  | CPR:6 | 151 | 0 | 5 | 6 | 108 | 0 |
|  | CPR:9 | 291 | 3 | 8 | 0 | 0 | 267 |

|  | Precision | Recall | F1-score | Support |
| --- | --- | --- | --- | --- |
| CPR:3 | 0.443 | 0.346 | 0.389 | 598 |
| CPR:4 | 0.592 | 0.545 | 0.567 | 1512 |
| CPR:5 | 0.544 | 0.385 | 0.451 | 161 |
| CPR:6 | 0.651 | 0.400 | 0.495 | 270 |
| CPR:9 | 0.483 | 0.469 | 0.476 | 569 |

# GRU

|  | Predicted | | | | | | |
| --- | --- | --- | --- | --- | --- | --- | --- |
| GS |  | NA | CPR:3 | CPR:4 | CPR:5 | CPR:6 | CPR:9 |
|  | NA | 8714 | 265 | 555 | 84 | 99 | 268 |
|  | CPR:3 | 257 | 268 | 69 | 1 | 1 | 2 |
|  | CPR:4 | 458 | 60 | 960 | 3 | 9 | 22 |
|  | CPR:5 | 71 | 2 | 2 | 82 | 4 | 0 |
|  | CPR:6 | 115 | 0 | 2 | 9 | 144 | 0 |
|  | CPR:9 | 313 | 5 | 21 | 0 | 0 | 230 |

|  | Precision | Recall | F1-score | Support |
| --- | --- | --- | --- | --- |
| CPR:3 | 0.447 | 0.448 | 0.447 | 598 |
| CPR:4 | 0.597 | 0.635 | 0.615 | 1512 |
| CPR:5 | 0.458 | 0.509 | 0.482 | 161 |
| CPR:6 | 0.560 | 0.533 | 0.546 | 270 |
| CPR:9 | 0.441 | 0.404 | 0.422 | 569 |

# ATT-RNN

|  | Predicted | | | | | | |
| --- | --- | --- | --- | --- | --- | --- | --- |
| GS |  | NA | CPR:3 | CPR:4 | CPR:5 | CPR:6 | CPR:9 |
|  | NA | 9033 | 134 | 574 | 79 | 47 | 118 |
|  | CPR:3 | 375 | 157 | 63 | 3 | 0 | 0 |
|  | CPR:4 | 579 | 39 | 884 | 4 | 3 | 3 |
|  | CPR:5 | 82 | 0 | 1 | 77 | 0 | 1 |
|  | CPR:6 | 162 | 0 | 0 | 4 | 104 | 0 |
|  | CPR:9 | 397 | 6 | 25 | 0 | 0 | 141 |

|  | Precision | Recall | F1-score | Support |
| --- | --- | --- | --- | --- |
| CPR:3 | 0.467 | 0.263 | 0.336 | 598 |
| CPR:4 | 0.571 | 0.585 | 0.578 | 1512 |
| CPR:5 | 0.461 | 0.478 | 0.470 | 161 |
| CPR:6 | 0.675 | 0.385 | 0.491 | 270 |
| CPR:9 | 0.536 | 0.248 | 0.339 | 569 |

# ATT-LSTM

|  | Predicted | | | | | | |
| --- | --- | --- | --- | --- | --- | --- | --- |
| GS |  | NA | CPR:3 | CPR:4 | CPR:5 | CPR:6 | CPR:9 |
|  | NA | 8919 | 195 | 524 | 55 | 61 | 231 |
|  | CPR:3 | 299 | 243 | 53 | 1 | 0 | 2 |
|  | CPR:4 | 547 | 58 | 889 | 4 | 3 | 11 |
|  | CPR:5 | 84 | 1 | 1 | 72 | 2 | 1 |
|  | CPR:6 | 130 | 0 | 3 | 3 | 134 | 0 |
|  | CPR:9 | 320 | 1 | 10 | 0 | 0 | 238 |

|  | Precision | Recall | F1-score | Support |
| --- | --- | --- | --- | --- |
| CPR:3 | 0.488 | 0.406 | 0.443 | 598 |
| CPR:4 | 0.601 | 0.588 | 0.594 | 1512 |
| CPR:5 | 0.533 | 0.447 | 0.486 | 161 |
| CPR:6 | 0.670 | 0.496 | 0.570 | 270 |
| CPR:9 | 0.493 | 0.418 | 0.452 | 569 |
